# Review of petroleum toxicity in marine reptiles

**DOI:** 10.1007/s10646-021-02359-9

**Published:** 2021-03-16

**Authors:** Elizabeth J. Ruberg, Tony D. Williams, John E. Elliott

**Affiliations:** 1 Department of Biological Sciences, Simon Fraser University, Burnaby, BC Canada; 2 Pacific Wildlife Research Centre, Environment and Climate Change Canada, Delta, BC Canada

**Keywords:** Petroleum toxicity, Marine reptiles, Physiology, Fitness, Mini-review, Sea turtles

## Abstract

Worldwide petroleum exploration and transportation continue to impact the health of the marine environment through both catastrophic and chronic spillage. Of the impacted fauna, marine reptiles are often overlooked. While marine reptiles are sensitive to xenobiotics, there is a paucity of petroleum toxicity data for these specialized fauna in peer reviewed literature. Here we review the known impacts of petroleum spillage to marine reptiles, specifically to marine turtles and iguanas with an emphasis on physiology and fitness related toxicological effects. Secondly, we recommend standardized toxicity testing on surrogate species to elucidate the mechanisms by which petroleum related mortalities occur in the field following catastrophic spillage and to better link physiological and fitness related endpoints. Finally, we propose that marine reptiles could serve as sentinel species for marine ecosystem monitoring in the case of petroleum spillage. Comprehensive petroleum toxicity data on marine reptiles is needed in order to serve as a foundation for future research with newer, unconventional crude oils of unknown toxicity such as diluted bitumen.

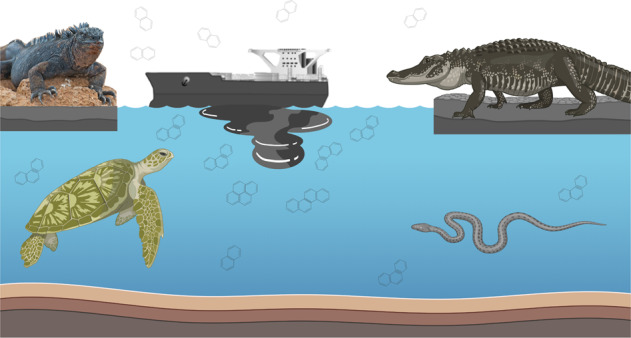

## Introduction

Petroleum spillage into the marine environment has detrimental impacts on all trophic levels of marine life (Murphy et al. 2016); while there is extensive literature regarding the impacts of petroleum to marine biota, reptiles have received very little attention. Though reptiles constitute nearly 30% of identified vertebrates worldwide, they were included in just 0.8% of published contaminant studies from 1996–2008 (Sparling et al. 2010). There are about 69 species of marine reptiles including over 60 species of sea snakes (not including at least 20 subspecies), 7 species of sea turtles, 1 marine iguana species (*Amblyrhynchus cristatus*), and 1 species of crocodile adapted to saltwater (*Crocodylus porosus*) although this crocodile tends to remain in brackish water (Rasmussen et al. 2011). However, only sea turtles have received more than the most cursory coverage (Sparling et al. 2000). Marine reptiles are important ecologically; for example, sea turtles play a key role in the balance of trophic webs (Sydeman et al. 2015; Bailey et al. 2017). Moreover, due to their ectothermic nature (Sydeman et al. 2015) marine reptiles have slow metabolisms and are consequently especially sensitive to xenobiotics (Silva et al. 2020). Within the complex mixture of organic compounds that form petroleum (Kennedy 2015; Ylitalo et al. 2017), the aliphatic hydrocarbons, naphthenic acids, monoaromatic hydrocarbons, and polycyclic aromatic hydrocarbons (PAHs) are contaminants of particular toxicological concern (Kennedy 2015). Low molecular weight PAHs partition to and tend to remain in water, where they are bioavailable for uptake by aquatic fauna including marine reptiles (Marsili et al. 2001).

The degree to which an oil spill could harm marine reptiles depends on the particular sensitivity of habitat or species present (National Academies of Sciences, Engineering, and Medicine 2016), wave action (Boudreau et al. 2009), and nature of oil released into the environment including density, viscosity, and solubility (Dupuis and Ucan-Marin 2015) amongst others. Oil type can predict the route by which toxicity is exerted. For example, if a light crude oil were to spill into the marine environment, inhalation of the aromatic fraction of volatile PAHs may cause acute mortality as observed in harbor seals (*Phoca vitulina*) following the *Exxon Valdez* oil spill (Frost et al. 1994; Peterson 2001). PAHs are known carcinogens and mutagens (Manzetti 2012) and in general light crude oils contain a higher proportion of two-ringed aromatic PAHs than heavy crude (Dupuis and Ucan-Marin 2015). Because reptiles such as marine turtles must continuously resurface for air (Hochscheid 2014) they could inhale toxic PAHs if resurfacing in an oil slick (Milton et al. 2003). Additionally, because marine turtles have extraordinary diving capacities (Hochscheid 2014), they may be at risk of prolonged exposure to aromatic hydrocarbons. If PAHs are inhaled before a deep dive, prolonged exchange between the blood and hydrocarbons could occur (Irving et al. 1941; Ridgway et al. 1969), and during deep dives it is possible that hydrocarbons may bypass the liver and directly enter the brain as they can for marine mammals (Geraci et al. 1989). The same may be true for marine iguanas, as they can dive as deep as 30 meters to graze on algae beds (Wikelski and Trillmich 1994).

Conversely, in the event of a heavy crude oil spill, oil could coat the external surface of marine reptiles due to its high viscosity and density (National Academies of Sciences, Engineering, and Medicine 2016), presenting a serious threat to survival (McDonald et al. 2017). For example, turtles can die from asphyxiation if oil smothers the respiratory surfaces (McDonald et al. 2017; Mitchelmore et al. 2017). Following the *Deepwater Horizon* oil spill, hundreds of marine turtles were coated in thick oil hindering their mobility and ability to thermoregulate (McDonald et al. 2017). Upon rescue those turtles were lethargic and body temperature was higher than usual (McDonald et al. 2017); heavily coated individuals would have died had they not been rescued (Mitchelmore et al. 2017).

While all marine reptiles are likely vulnerable to oil spills, sea turtles in particular share their habitat with anthropogenic oil exploration, oil transportation, and oil development. This is demonstrated by increasing reports of marine turtles harmed by oil in the Gulf of Mexico, the Atlantic, the Caribbean Sea, the Gulf of Iraq, the Mediterrean, and the Red Sea (Lutcavage et al. 1997). All marine turtles are protected under the 1973 U.S. Endangered Species Act, listed as either threatened or endangered (Milton et al. 2003; Zychowski and Godard-Codding 2017). Additional protection in Canada includes the listing of leatherbacks (*Dermochelys coriacea*) and loggerheads (*Caretta caretta*) as endangered (COSEWIC 2010, 2012). Increasing petroleum transportation in sea turtle habitat (Yender and Mearns 2003) and associated spillage places pressure on those populations already in decline (Yender and Mearns 2003; Lauritsen et al. 2017). Petroleum and tar contaminate extensive areas of ocean surfaces, such as the Sargasso Sea which entraps approximately 70,000 metric tons of tar due to convergent zones and drift lines (Lutcavage et al. 1997; Milton et al. 2003). Likewise, Sargassum communities, important habitat for juvenile sea turtles, accumulate floating oil (McDonald et al. 2015) and turtle nesting beaches and nearshore habitats are often at risk of oil pollution (Milton et al. 2003; Ylitalo et al. 2017). The distribution of marine turtles offshore of the Gulf of Mexico and the western Atlantic is linked to zones of convergence (Raloff 1986; Lutcavage et al. 1997), as Langmuir cells and convergence fronts accumulate prey consumed by turtles such as zooplankton. However, by the same mechanism, these feeding zones also attract debris, oil, and tar, increasing the likelihood of oil exposure to pelagic foragers such as marine turtles (Hall et al. 1983; Raloff 1986; Lutcavage et al. 1997). Consequently, marine turtles at all life stages are at risk of exposure to petroleum, whether through through direct contact or by contamination of their habitats (Lutcavage et al. 1997). Because each life stage will frequent a habitat where there is possibility of petroleum contamination, marine turtles are at a high degree of risk (Milton et al. 2003).

Similarly, marine iguanas have a vulnerable status (Macleod et al. 2020). An estimated 20–30% of the total marine iguana population is in decline due to invasive species, and petroleum pollution presents an additional threat to those populations (Macleod et al. 2020). Additionally, the marine iguana is found only in the Galapagos islands (Macleod et al. 2020). While mainly terrestrial, it forages on algae in the intertidal zone or dives to access algal beds up to 30 m below sea surface (Wikelski and Trillmich 1994). As a result, the marine iguana is at risk of exposure to petroleum hydrocarbons through foraging activities such as grazing and diving. While the marine iguana is indeed vulnerable to petroleum contamination, we retrieved only three papers documenting the exposure of marine iguanas to a single oil spill (Wikelski et al. 2001, 2002; Romero and Wikelski 2002).

Among sea snakes, 27 species are listed on the IUCN Red List as vulnerable (16 species), endangered (7 species), or critically endangered (4 species) (IUCN 2020). Sea snakes are distributed throughout the tropical and sub-tropical waters of the Pacific and Indian Oceans and threatened by commercial exploitation including sea snake fisheries and bycatch (Rasmussen et al. 2011). However, their conservation is limited due to an incomplete knowledge of taxonomy, breeding cycles, and general demography, particularly in Asia (Rasmussen et al. 2011). Sea snakes are likely vulnerable to oil spills as they swim and dive in the open ocean, commonly preying on fish that are generally bottom-dwelling, while a few species consume mollusks and crustaceans (Voris 1972). Consequently, sea snakes could come into contact with oil slicks or petroleum hydrocarbons by swimming, diving, contacting contaminated sediments, or consuming mollusks and crustaceans that are known to bioconcentrate petroleum hydrocarbons. Unfortunately, we found no data regarding the impact of petroleum to sea snakes in our literature search. Just two papers reported PAH concentrations detected in sea snake tissue (Sereshk and Bakhtiari 2014; Mote et al. 2015).

The saltwater crocodile inhabits the rivers, lakes, swamps, marshes, and coastal brackish waters of northern Australia, eastern India, and Southeast Asia (Crocodile Specialist Group 1996). It is listed as least concern by the IUCN; however, this species was last assessed in 1996, consequently re-assessment is needed (Crocodile Specialist Group 1996). The largest of the reptiles, the saltwater crocodile has been recorded to reach over 6 meters long (Montague 1983). While females generally nest near freshwater rivers and swamps (Harvey and Hill 2003), juvenile crocodiles are often forced out of their river habitat by large territorial males into coastal marine areas (Semeniuk et al. 2011) or into the ocean (Rasmussen et al. 2011). They have also been commonly reported swimming in the open ocean for great distances (Manolis 2005). While saltwater crocodiles consume a variety of prey, they have been observed on the beaches of northern Australia preying on the flatback (*Natator depressus*) and Olive ridley (*Lepidochelys olivacea*) sea turtles, specifically nesting females, hatchlings, and eggs (Whiting and Whiting 2011). Consequently, saltwater crocodiles could be vulnerable to oil spills when swimming in the open ocean and could come into contact with petroleum on oiled beaches when hunting for turtle eggs, nestlings, and females. Unfortunately, our Web of Science™ search did not retrieve any papers on the toxicity of petroleum to the saltwater crocodile. Nevertheless, crocodiles have been reported to be in the vicinity of oil spills in Australia (Lipscombe 2000), while two individuals were reported oiled in the mangrove forests of Bangladesh from a nearby oil spill (Chowdhury and Akber 2015). However, those reports did not specify the crocodile species affected.

Because we found little to no data on the impacts of petroleum to sea snakes, marine iguanas, and saltwater crocodiles, this literature review will focus on petroleum toxicity in marine turtles with a small section on the impact of oil spillage to a population of marine iguanas. The following impacts of petroleum toxicity are divided into fitness-related, physiology-related, and population level effects.

## Methods

The literature search was completed in Web of Science™ (accessed May 2018 and July 2020). All years from 1900 to 2020 were included with no filters used. Search terms included a petroleum derivative, vertebrate class, and any words including toxic. Petroleum derivatives used in the search were “petroleum” OR “fuel” OR “hydrocarbon” OR “oil spill” OR “bitumen” OR “crude oil.” Vertebrate search terms included “reptile.” Specifically, the Boolean phrase was “TS = ((petroleum OR fuel OR hydrocarbon OR “oil spill” OR bitumen OR “crude oil”) AND (*reptile*) AND (*toxic*))”. For the purposes of this paper ‘fitness’ is defined as survival or mortality and reproductive events while ‘physiological effects’ are defined as effects on the cell, tissue, or organ level of marine vertebrates. Including search terms such as “physiology”, “endpoint”, “reproduction”, and “fitness” to the original Boolean phrase was not useful. Few (two) relevant marine reptile papers were found through the Web of Science™ search. Consequently, we included information from the following sources: select references within papers retrieved from the search (if accessible), information from a Canadian Department of Fisheries and Oceans report (Dupuis and Ucan-Marin 2015), one book chapter (Frasier et al. 2020), and recent *Deepwater Horizon* spill papers that had not been retrieved through Web of Science™. In this way, we have tried to represent oil spill research on marine reptiles as objectively and accurately as possible. Therefore, this review is based on information from over 40 papers related to conventional petroleum impacts on marine reptiles.

## Overview

Marine turtles are exposed to oil through multiple routes. Sea turtles rapidly inhale air from the surface layer before diving, allowing petroleum vapor into the lungs, considered the most acutely harmful route of exposure (Lutcavage et al. 1997; Milton et al. 2003). In addition, sea turtles may continuously resurface through oil slicks and have been observed to do so for up to an hour (Lutcavage et al. 1997), resulting in prolonged physical contact with oil and fouling of the eyes, nasal cavaties, mouth, and tongue (Lutcavage et al. 1995; Milton et al. 2003; Ylitalo et al. 2017). Consumption of food contaminated with oil, consumption of invertebrates in which oil hydrocarbons have bioconcentrated, or consumption of oiled sediments during feeding enables intake of oil, particularly toxic hydrocarbons such as PAHs into the gut and intestine. Consequently, risk of oil hydrocarbon ingestion differs according to each species’ feeding habits (Milton et al. 2003). Kemp’s ridleys (*Lepidochelys kempii*) and loggerheads primarily consume crustaceans and molluscs, which can bioconcentrate PAHs from oil, and are consequently at higher risk of exposure to PAHs than the herbivorous green turtle (*Chelonia mydas*) (Milton et al. 2003). Oil pollution may also reduce food availability, such as when a 1986 oil spill caused intertidal beds of turtle grass (*Thalassia testudinum*) to die, wiping out the main food source of green turtles (Milton et al., 2003).

Marine turtles have low digestive rates typical of ectotherms, and oil does not pass rapidly through the gut; consequently, the probability of PAH absorption is increased (Milton et al. 2003). Oil in or on nesting sites may also directly interfere with embryonic development via eggshell exposure (Lutcavage et al. 1997), and turtles at early life stages are at greatest risk of oil toxicity (Milton et al. 2003). Oiling may also impact turtle populations by altering sex distribution of hatchlings (Hays et al. 2001). Oil washed onto nesting beaches may change sand albedo and consequently alter the temperature of egg incubation, an important parameter determining sex of hatchlings, consequently sex ratios could be altered (Hays et al. 2001). To summarize, for marine turtles, the routes of exposure to oil encompass aspiration of volitiles, dermal contact, ingestion of oil or of prey in which PAHs have bioconcentrated, oil fouling of sense organs, and oiling of eggs on nesting beaches (Milton et al. 2003; Mitchelmore et al. 2017; Ylitalo et al. 2017).

Small petroleum spills are frequent and prevalent globally (Anderson and LaBelle 1994), thereby increasing exposure of marine reptiles to PAHs. Among 367 loggerheads from the Canary Islands and Cape Verde blood sampled from 2007 to 2010, baseline total PAH concentrations were around 5 ng/ml (0.005 ug/ml) (Camacho et al. 2012). This is well below the range reported for acute toxicity in aquatic organisms (0.2–10 ug/g), but within the range that may elicit sublethal effects (0.005-0.01 ug/g) (Neff 1985). Phenanthrene, a peterogenic PAH dominant in weathered crude oil, was most commonly detected, indicating exposure to oil from small spillages (Camacho et al. 2012). Consequently, it is likely that sea turtles are subject to chronic exposure of oil hydrocarbons (Camacho et al. 2012) that may cause sublethal effects in individuals as well as populations.

Four relevant laboratory studies involving marine reptiles were retrieved in this review (Fritts and McGehee 1982; Lutcavage et al. 1995; Rowe et al. 2009; Harms et al. 2019). Those studies were confined to investigating sublethal effects, and required to be fully reversible once the exposure terminated due to the protected nature of marine turtles (Milton et al. 2003), (note: Rowe et al. (2009) used a freshwater turtle as a surrogate). Aside from those few laboratory studies, little else is known regarding the toxicological effects of oil to marine turtles. Strandings due to ingestion of or adherence of tar balls to turtles are reported, along with sea turtle oilings. Reports of oiled turtles following a spill are often anecdotal (Lutcavage et al. 1997; Milton et al. 2003; Shigenaka et al. 2003; Yender and Mearns 2003). While this may be due in part to inconsistent reports of turtles as resources at risk during spill response efforts, the low probability of observation or recovery of oiled turtles due to their large home ranges, seasonal movements and long range migratory behavior is also a factor (Lutcavage et al. 1997; Yender and Mearns 2003). As knowledge regarding the effects of oil on marine turtles is incomplete (Stacy et al. 2017), freshwater testudines such as the snapping turtle (*Chelydra serpentina*) have been utilized as surrogate species in toxicity experiments (Rowe et al. 2009).

Following exposure to oil, turtles are cleaned with mild detergent (3% solution) on all superficial areas (Saba and Spotila 2003; Stacy et al. 2017) or with oil sorbent sheets (Lutcavage et al. 1995). When multiple cleanings are required, in addition to mild detergent, vegetable oil, and mayonnaise can assist (Stacy et al. 2017). Following rescue from the Gulf of Mexico, *Deepwater Horizon* oil-coated turtles were orally administered menhaden oil or cod liver oil with mayonnaise (Stacy et al. 2017). Standard treatment for rescued turtles can also include administration of fluids with vitamin B complex, iron, calcium supplementation, and when needed, antibiotics (Stacy et al. 2017).

## Physiological effects of exposure from laboratory studies

A range of physiological effects resulted when post-hatchling loggerhead turtles were kept for 96 hours in a tank with a 0.5 mm thick South Louisiana crude oil slick which they had to pass through repeatedly to breathe (Lutcavage et al. 1995). These included skin sloughing on the neck, flippers, and mucous membranes, changes in diving patterns and respiration, changes in energy metabolism, an increase in white blood cells, and infiltration of inflammatory cells in the epidermis (Lutcavage et al. 1995, 1997). Histologic changes in the skin included proliferation of pre-cancerous, abnormal, and inflamed skin cells (Milton et al. 2003). An incidental finding included the failure of the salt glands to function in two post-hatchlings (Lutcavage et al. 1995, 1997) which was possibly stress-related (Reina and Cooper 2000). After the 96 hour exposure, most physiological effects resolved within three weeks (Lutcavage et al. 1995). As recovery of the epidermis took up to three weeks (Milton et al. 2003), adverse effects on the epidermis elude to possible skin infections in oil-exposed turtles in the field (Lutcavage et al. 1995). Lutcavage et al. (1997) concluded that acute exposure to South Louisiana crude oil elicits adverse effects on nearly all the major physiological systems in sea turtles.

An additional exposure with post-hatchlings investigated the effects of exposure to oil, dispersant, or an oil/dispersant combination for 1 or 4 days (Harms et al. 2019). The treated loggerhead hatchlings exhibited numerous significant changes in clinical pathology parameters including significantly higher packed cell volume (PCV) in combination exposure groups (Harms et al. 2019). Hatchlings in the oil and combination group (1 day and 4 day treatments) as well as the dispersant group (4 day treatment) also failed to gain weight (Harms et al. 2019) as compared to control hatchlings. Lastly, elevated heterophil/lymphocyte ratios and higher estimated heterophil and leukocyte counts in the combination and oil exposure groups, as well as elevated plasma glucose in the dispersant and combination groups were suggestive of a hypothalamic-pituitary-adrenal (HPA) stress response (Harms et al. 2019).

## Fitness effects of exposure from laboratory studies

After the 1979 *Ixtoc* *I* blowout in the Gulf of Mexico, Fritts and McGehee (1982) conducted an in situ field study on Kemp’s ridley nesting sites oiled by the spill. Hatching success and mortality were monitored on both oiled and unoiled beaches; there was no significant effect of oiled sand on hatching success of Kemp’s ridley sea turtles, likely because the oil was well weathered by wave action. However, to follow up this negative result, a laboratory study was conducted by the same authors (Fritts and McGehee 1982) using loggerhead turtle eggs incubated in sand collected from the field. Fresh Louisiana crude oil was either poured overtop of sand in which eggs were incubating or mixed with sand in which eggs were incubating. Experimental results indicated a treatment effect on incubation time, hatchling morphology, number of hatchlings surviving to release, number of unhatched eggs, and number of unhatched eggs with embryos, contrasting with the field data (Fritts and McGehee 1982). Weathered oil was less harmful to developing embryos than fresh oil (Douben 2003) and oil poured overtop of sand in which eggs were incubating significantly increased mortality as compared to those incubating in sand mixed with oil (Milton et al. 2003).

Rowe et al. (2009) used eggs of the freshwater snapping turtle as a surrogate for marine turtle eggs (due to similar egg structure) with an exposure to an Arabian light crude oil/dispersant combination or Arabian light crude oil alone, at 5 or 10 grams oil /litre freshwater. The single exposure simulated environmental conditions by passing the oil or oil/dispersant combination through nest substrate to reach the eggs. Hatchlings were monitored until study termination at 13 months old. Eggs accumulated up to 560 µg/kg total PAHs; however, there was a lack of overall biological effects on hatchlings including survival, DNA integrity, growth, metabolism, energy storage, and behavior (Rowe et al. 2009). Together, the Rowe et al. (2009) and Fritts and McGehee (1982) studies demonstrate that both weathering and nesting substrate mediate the toxicity of oil or oil/dispersant combinations to turtle eggs (Zychowski and Godard-Codding 2017).

Hatching success requires optimal gas exchange, nest temperature, and moisture, all of which can be compromised by oil exposure (Milton et al. 2003). Oil may fill the interstitial spaces within the nest, preventing diffusion of oxygen from sand to eggs, shifting the balance of moisture within the nest or may interfere with nest temperature by shifting the color of the sand and consequently thermal conductivity (Milton et al. 2003). When petroleum jelly was painted on eggs of flatback and green turtles, it reduced gas exchange and embryo survival (Phillott and Parmenter 2001). Survival of the embryo was altered according to location of application, proportion of egg covered, and species sensitivity (Phillott and Parmenter 2001). Consequently, in a spill scenario, regardless of toxicity, oil may physically smother embryos and reduce survival rates of nests in the field (Milton et al. 2003).

## Physiological effects from oil spill studies

Approximately 540 sea turtles were rescued during the *Deepwater Horizon* oil spill, of which 456 were visibly oiled (Harms et al. 2014). Of those, 319 oiled marine turtles (60% Kemp’s ridley, 35% green turtles, 3% hawksbills (*Eretmochelys imbricata*), and 2% loggerheads) were sent to rehabilitation facilities, and all but 4 survived. Anemia (PCV < 20%) was reported in 7% of these sea turtles, but that was reportedly not due to oil exposure (Stacy et al. 2017). Once rescued turtles were received in the rehabilitation center, a range of clinical endpoints were examined; however, no petroleum-related acute toxicological impacts were reported. Any clinicopathological abnormalities observed were attributed to stress, exertion, physical exhaustion, and dehydration as a result of physically being covered in oil or due to capture and transport (Stacy et al. 2017).

Further field-related impacts to sea turtles can arise. The International Convention for the Prevention of Pollution from Ships (MARPOL) regulates disposal of oil at sea, such as routine operations like disposal of oily engine room wastes (Walker et al. 2019). While illegal discharge of oil at sea is monitored by satellite in Canada, the United States, and France, gaps in enforcement remain (Vollaard 2017). “Tar balls,” a by-product of ship operations, are created from bilge tank flushing and tank washings, and of natural coastal oil seeps (Milton et al. 2003). Convergence zones and Langmuir cells will aggregate tarballs in feeding zones utilized by sea turtles, specifically where post-hatchlings feed (Shigenaka et al. 2003). Chemical analysis of tarballs that were ingested by loggerheads found in the Atlantic coast offshore Florida determined the tar was derived from numerous sources (Witherington 2002). Consequently, tarballs are ubiquitous across oceans and beaches (Milton et al. 2003) and represent various petrogenic sources.

Sea turtles do not appear to avoid oil or tarballs (Gramentz 1988), they cannot differentiate between tarballs and food (Gramentz 1988; Zychowski and Godard-Codding 2017) and, in fact, they actively ingest floating oil (Van Vleet and Pauly 1987). Among loggerheads caught as bycatch in the Maltese Islands, 17% were either oiled or had ingested oiled substrates or tarballs (Gramentz 1988) while 34% of post-hatchlings and 20% of neonate turtles captured in the Atlantic near Florida contained tarballs in their stomach and esophagi (Witherington 2002). Upon ingestion, tarballs may cause starvation via physical blockage in the gut or by decreasing absorption efficiency. In addition they may increase absorption of oil toxicants, cause necrosis, ulceration, interfere with the metabolism of fats, or cause buoyancy problems (Milton et al. 2003). Therefore, tarballs are a threat to marine turtles, specifically those at the post-hatchling and juvenile stages.

## Fitness effects from oil spill studies

Most sea turtles oiled in the wild go unreported. Turtle carcasses are prone to sinking (Epperly et al. 1996); moreover, the large home ranges and long range migratory behavior of sea turtles limit probability of detection (Lutcavage et al. 1997; Yender and Mearns 2003). Of those sea turtle strandings that were reported in Florida, only 3% were petroleum related (Vargo et al. 1986). Yearly rates of strandings due to oil were higher on Texas beaches (up to 6%) and in Dade County, Florida (37.5%; 1980-84); but overall in the United States. only about 1% of turtle strandings reported were related to oil contamination (Lutcavage et al. 1997). On the coast of Brazil, less than 1% of stranded or dead green turtles were oiled (Bugoni et al. 2001). In contrast, in the Canary Islands of Spain, from 1998–2011, 3.1% of loggerhead turtle strandings were due to oil contamination (Camacho et al. 2013). Interestingly, most reports of oiled turtles in the NOAA HAZMAT incidents database from 1977–2001 were due to spills from smaller vessels, pipelines, and dock facilities rather than catastrophic tanker spills (Yender and Mearns 2003).

Confirmation that turtles were affected by oil spills occurred following the 1979 *Ixtoc* *I* oil spill (Zychowski and Godard-Codding 2017). Six turtles were treated and 3 heavily oiled turtles died (Hall et al. 1983). Necropsies revealed chronic oil exposure in all individuals (Hall et al. 1983). During the 1983 *Nowruz* oil spill in the Persian Gulf, there were at least 180 hawksbill turtle mortalities (Lutcavage et al. 1997). Miller (1989) estimated that over 500 hawksbill and over 500 green turtles died, wiping out nearly the entire hawksbill population and the majority of the green turtle population in that region. Furthermore, petroleum spills have varying impact on nesting beach sites. Following the barge *Bouchard B155* 1993 spill, approximately 200 turtle hatchlings died, while it was estimated that both oil spill clean up and petroleum exposure may have harmed an additional 2000 or more (Yender and Mearns 2003). Following that spill, two nests were covered in oil and were estimated to have a 5% hatching rate as compared to the standard 50–90% hatching rate (Florida Department of Environmental Protection, National Oceanic and Atmospheric Administration, U.S. Department of the Interior 1997). In contrast, a turtle nesting site in Florida normally producing around 137,000 loggerhead, green, and leatherback hatchlings was contaminated by a spill in 2000; National Resource Damage Assessment modeling estimated the spill caused approximately 7800 mortalities, reducing hatchling production by 0.06% (Yender and Mearns 2003). Lastly, a before-after control-impact statistical modeling approach was used to estimate the impact of the *Deepwater Horizon* spill on loggerhead nest densities in NW Florida beaches heavily affected, estimating that during the 2010 nesting season, nest densities were reduced by 43.7% (95% CI: 10–65%), (Lauritsen et al. 2017). In summary, a number of spills have been documented to affect both individual sea turtles and local nesting grounds, with varying degree of impact. Beach clean-up operations following spills may contribute to hatchling mortality, which should be avoided if at all possible.

Following the 2010 *Deepwater Horizon* oil spill in the Gulf of Mexico, over 1000 marine turtles were collected, including dead oiled turtles, live oiled turtles, and dead unoiled turtles presumed to have died from the spill (U.S. Fish and Wildlife Service 2011; Barron 2012). Of 536 sea turtles rescued, 85% were visibly oiled (Harms et al. 2014), 319 oiled turtles were sent to rehabilitation facilities and 99% of those were successfully released (Stacy et al. 2017). In all, 97% of external oil samples from marine turtles were identified as *Deepwater* *Horizon* oil (Ylitalo et al. 2017), and marine turtles in the Gulf of Mexico were exposed to oil through multiple exposure routes (Ylitalo et al. 2017). All life stages were at risk of exposure, as marine habitats, nearshore habitats, and nesting sites were potentially affected (Milton et al. 2003; Ylitalo et al. 2017). However, because search teams did not survey the entire gulf, and were only active for 4 months, it was not known how many mortalities actually resulted (Mitchelmore et al. 2017).

McDonald et al. (2015) estimated that tens of thousands of sea turtles were affected by the *Deepwater Horizon* oil spill as they were likely in range of the spill but not rescued or otherwise encountered. It has also been suggested that a significant portion of turtles that were not rescued likely succumed to mortality (Stacy et al. 2017). Based on blood chemistry abnormalities associated with risk of mortality in a prognostic model, over 50% of rescued turtles would have been at risk of mortality if they had not been rescued (Stacy et al. 2017). Additionally, a sea turtle technical working group concluded that 100% of heavily oiled turtles would have died from being physically oiled in high temperatures; however, an additional 266 were minimally oiled, 87 lightly oiled, and 47 moderately oiled (Stacy 2012; DWH Trustees 2015; Wallace et al. 2017; Mitchelmore et al. 2017). Using multiple lines of evidence, Mitchelmore et al. (2017) estimated mortality of those sea turtles categorized as minimally, lightly, and moderately oiled. They concluded 30% of those sea turtles would have died from ingestion of oil and even minimally oiled turtles were at risk for adrenal insufficiency, which could lead to long term adverse effects on the survival and fitness of populations (Mitchelmore et al. 2017). In summary, these modeling approaches suggest that mortality based on recoveries of dead oiled turtles markedly underestimates the impact of oil pollution.

While dispersants were used following the *Deepwater* *Horizon* spill, they were below detection levels in external oil samples from rescued turtles, and only one necropsy revealed traces of the dispersant component dioctyl sodium sulfosuccinate in the esophagus (Ylitalo et al. 2017). Dispersants may increase bioavailability of petroleum hydrocarbons in fish embryos (Adams et al. 2014) or interact additively or synergistically with oil in zooplankton (Almeda et al. 2014) which are prey of turtles. While it is unknown what effect the dispersant/*Deepwater Horizon* oil combination had on Gulf of Mexico marine turtles, Harms et al. (2019) indicated oil/dispersant combinations can cause osmolarity, electrolyte, and hydration abnormalities during acute exposure and interfere with hatchling growth in loggerheads.

## Linking physiology, fitness, and populational level effects—a case study

Petroleum contamination near the Galapagos Islands had a large impact on a population of marine iguanas (*Amblyrhynchus cristatus*) (Wikelski et al. 2001, 2002; Romero and Wikelski 2002). This account of the following oil spill is the only example found in this review that linked fitness, physiology, and population level impacts together. Based on long term monitoring, the population of 170 marine iguanas on Santa Fe island was reduced by 62% (*P* < 0.001) a year after a nearby oil spill (Wikelski et al. 2002). The petroleum, a mixture of diesel and bunker, was dispersed by strong currents, consequently waters reached 44 ppm oil, or low level contamination (Wikelski et al. 2002). Previous studies showed that high corticosterone levels in this marine iguana population linearly correlated with mortality (Romero and Wikelski 2002). Following the spill, 70% conspecifics on Santa Fe island were externally oiled (Wikelski et al. 2001), but there were no immediate deaths. However, seven days after the spill, iguanas that were both visibly oiled and unoiled had elevated plasma corticosterone levels, as compared to pre-spill samples (Wikelski et al. 2001) predicting a 40% mortality rate (Romero and Wikelski 2002). Eleven months later, the population had been reduced by 62%, inferring that corticosterone levels observed one week after the spill had predicted the long-term population effects of the oil (Romero and Wikelski 2002). Because high corticosterone levels promote protein catabolism (Dallman et al. 1993), and the iguana diet consisted of algae, the iguanas’ gut bacteria that aided in digestion of algae cellulose was likely eliminated by oil (Romero and Wikelski 2002). If so, the iguanas would not have been able to obtain the algae’s nutrients, causing a rise in corticosterone levels, inducing protein catabolism, and eventually starvation (Romero and Wikelski 2002).

## Summary and recommendations

Oil spills have provided the context for many studies included in this review, since the protected status of marine reptiles negates acute dosing with oil in laboratory studies (Zychowski and Godard-Codding 2017). Alternative in vitro techniques have been developed with the potential for toxicological use in reptilian species: the development and preserving of marine turtle embryo cell cultures (Moore et al. 1997) and harvesting of primary cell cultures from loggerhead turtles, the latter successfully used to assess the induction of the enzyme cytochrome P450 1A (CYP1A) with benzo[a]pyrene (Webb et al. 2014). However, more research is needed on development of biomarkers of exposure and the specific toxicological effects elicited by the constituents of oil, such as PAHs, as current research indicates differences in specificity within reptiles (Zychowski and Godard-Codding 2017).

Oil can harm marine turtles following both internal and external exposure. In particular, lesions are a common theme. Discovery of internal lesions related to esophageal impaction, necrotizing gastroenteritis, necrotizing hepatitis, and tubulonephrosis were found in turtles that had died from petroleum exposure (Camacho et al. 2013). The skin damage observed in the Lutcavage et al. (1995) study including lesions, skin sloughing, inflamed, abnormal, and dead skin cells in marine turtles, resulting from external exposure to petroleum, presented a potential route of entry for pathogens. Mild skin and eye irritation was also observed in oil-exposed freshwater turtle species (Saba and Spotila 2003). While skin lesions were not reported for marine iguanas, over half of the 170 individuals examined had oil residue on their skin (Wikelski et al. 2001). Lesions were also commonly documented in fishes following the *Deepwater* *Horizon* oil spill (Frasier et al. 2020). The high frequency of skin lesions in Gulf of Mexico benthic fish was hypothesized to be from PAH suppression of the immune system, whereby opportunistic bacteria then colonized (Murawski et al. 2014; Frasier et al. 2020). A similar mechanism may occur in marine reptiles.

Additionally, based on the evidence that petroleum increases corticosterone levels in marine iguanas, and the suggested HPA stress response in turtle hatchlings following petroleum exposure (Harms et al. 2019), it is likely that petroleum affects the HPA axis in marine reptiles. This is possibly because the adrenal gland can be a significant site for metabolism of PAHs (Venn-Watson et al. 2015). Likewise, exposure to petroleum increases risk of adrenal insufficiency in marine turtles (Mitchelmore et al. 2017). The increased corticosterone levels and severe population level impact of low level petroleum contamination to marine iguanas (Wikelski et al. 2001, 2002; Romero and Wikelski 2002) demonstrates the sensitivity of marine iguanas to petroleum. Similar to marine iguanas, herbivorous marine turtles such as the green turtle also contain symbiotic bacteria in the gut aiding in digestion of plant materials like cellulose. Consequently, they may be equally susceptible and sensitive to low-level oil contamination (Milton et al. 2003). Because of their sensitivity, marine reptiles could serve as sentinel species for marine ecosystem monitoring, specifically for petroleum spillage.

**Fig. 1:**
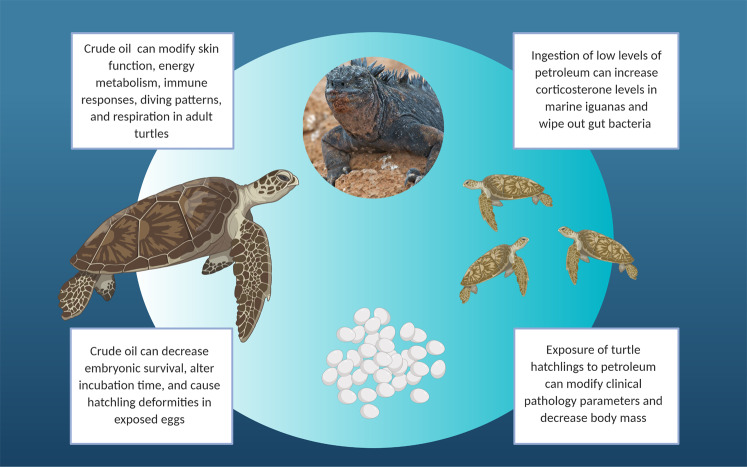
Summary of the common impacts of petroleum toxicity to marine reptiles

Currently, many knowledge gaps remain. Existing knowledge of petroleum’s impacts on marine turtles is based on limited toxicity studies (Fritts and McGehee 1982; Lutcavage et al. 1995; Harms et al. 2019). Those studies showed evidence of petroleum altering incubation time, morphology, hatchling survival, clinical chemistry and hematological parameters, weight, skin function, metabolism, immune responses, diving patterns, and respiration (Fig. 1). Aside from the aformentioned studies, there is a paucity of acute and chronic petroleum toxicity tests in the literature. Additionally, the toxic mechanisms of crude oil and its components on the physiological systems of sea turtles and other marine reptiles are understudied. Determination of oil toxicity is further complicated following rescue of oiled turtles as health assessment results are difficult to disentange from stress due to handling, captivity, and changes in clinical parameters due to starvation (Stacy et al. 2017). Additionally, while the routes of petroleum exposure to marine turtles have been well reviewed (Lutcavage et al. 1997; Shigenaka et al. 2003; Mitchelmore et al. 2017), overviews of the routes of exposure by which petroleum impacts marine iguanas, sea snakes, and saltwater crocodiles are lacking in the literature.

It is difficult to directly quantify marine turtle mortalities following petroleum spillage. Because turtle carcasses are prone to sinking (Epperly et al. 1996), the observed acute effects of petroleum are unlikely to be accurate following an oil spill. However, modeling approaches based on both direct observations of oiled reptiles and satellite-derived surface oil distributions can be helpful in estimating the probability of oil exposure for reptiles present within an oil-spill footprint (Wallace et al. 2017).

Furthermore, petroleum’s chronic impacts on marine turtles are undetermined, even though marine turtles are long-lived species (Kocmoud et al. 2019). The chronic effects of *Deepwater Horizon* oil to marine turtles in the Gulf of Mexico are still unknown (Vander Zanden et al. 2016; Frasier et al. 2020). Collection of keratin samples from loggerhead turtles combined with the use of satellite tracking indicated that loggerheads in the northern Gulf of Mexico remained in oil-contaminated sites, exhibiting high site fidelity following the spill (Vander Zanden et al. 2016). Additionally the critically endangered Kemp’s ridley turtle nests and completes its lifecycle primarily in the Gulf of Mexico (Kocmoud et al. 2019). The Kemp’s ridley mortality event that coincided with the 2010 spill and resulting nest decline in 2010 (35% decrease as compared to 2009) and 2013 (Gallaway et al. 2016) may be a result of the long-term impacts of oil. However, nest reductions may also be influenced by population dynamics such as increased population size coupled with a reduction in prey sources (Gallaway et al. 2016). Annual nest counts at the Kemp’s ridley primary nesting beach indicate the number of nests have been increasing since 2015 (Kocmoud et al. 2019). Age-structured population modeling has been used to investigate cause-effect relationships between Kemp’s ridley population dynamics and the *Deepwater Horizon* spill (Kocmoud et al. 2019), but the long term effects of the spill remain enigmatic. However, it is clear that both loggerheads and Kemp’s ridleys experienced chronic exposure to *Deepwater Horizon* oil.

We recommend that researchers continue to use species such as the freshwater snapping turtle as surrogates for marine turtles in petroleum toxicity studies. For example, the 14 day crude oil exposures to red-eared sliders and common snapping turtles (Mitchelmore et al. 2015) and the previously mentioned exposure of petroleum and dispersant to freshwater snapping turtle eggs (Rowe et al. 2009). In addition, petroleum toxicity tests must be conducted on sea snakes and appropriate surrogates for marine iguanas and saltwater crocodiles. Most importantly, petroleum toxicity tests must be standardized. We suggest a single species or surrogate be utilized across all toxicity tests per marine reptile taxa. In addition, dosage of crude oil administered should be consistent across a range of crude oils evaluated, and acute and chronic durations must be standardized by day. Standardized toxicity data will allow for extrapolation to petroleum toxicity in marine reptiles and clarify the mechanisms by which marine reptile mortalities occur in the field following petroleum spillages, linking physiological effects to fitness related impacts.

More widespread application of non-invasive field techniques such as collection of keratin samples from the carapace of sea turtles can assist in quantifying the long term foraging history of marine reptile populations in relation to oil spill footprints (Vander Zanden et al. 2016). This, as well as the use of satellite tracking via attachment of satellite transmitters to individuals (Vander Zanden et al. 2016) such as sea turtles and saltwater crocodiles could greatly assist in collection of both pre-spill and post-spill data, allowing for determination of population level impacts.

Increasing transportation of unconventional crude oils such as diluted bitumen across North America or in large volumes by tanker poses new concern for marine fauna. Comprehensive petroleum toxicity data on marine reptiles is needed in order to serve as a foundation for future research with unconventional crude oils of unknown toxicity such as diluted bitumen.
